# Genomic selection requires genomic control of inbreeding

**DOI:** 10.1186/1297-9686-44-27

**Published:** 2012-08-16

**Authors:** Anna K Sonesson, John A Woolliams, Theo HE Meuwissen

**Affiliations:** 1 Nofima AS, 1431 Ås, Norway; 2 University of Life Sciences, Department of Animal and Aquacultural Sciences, Nofima 1432 Ås, Norway; 3 The Roslin Institute and R(D) SVS, University of Edinburgh, Roslin, Midlothian, EH25 9PS, UK

## Abstract

**Background:**

In the past, pedigree relationships were used to control and monitor inbreeding because genomic relationships among selection candidates were not available until recently. The aim of this study was to understand the consequences for genetic variability across the genome when genomic information is used to estimate breeding values and in managing the inbreeding generated in the course of selection on genome-enhanced estimated breeding values.

**Methods:**

These consequences were measured by genetic gain, pedigree- and genome-based rates of inbreeding, and local inbreeding across the genome. Breeding schemes were compared by simulating truncation selection or optimum contribution selection with a restriction on pedigree- or genome-based inbreeding, and with selection using estimated breeding values based on genome- or pedigree-based BLUP. Trait information was recorded on full-sibs of the candidates.

**Results:**

When the information used to estimate breeding values and to constrain rates of inbreeding were either both pedigree-based or both genome-based, rates of genomic inbreeding were close to the desired values and the identical-by-descent profiles were reasonably uniform across the genome. However, with a pedigree-based inbreeding constraint and genome-based estimated breeding values, genomic rates of inbreeding were much higher than expected. With pedigree-instead of genome-based estimated breeding values, the impact of the largest QTL on the breeding values was much smaller, resulting in a more uniform genome-wide identical-by-descent profile but genomic rates of inbreeding were still higher than expected based on pedigree relationships, because they measure the inbreeding at a neutral locus not linked to any QTL. Neutral loci did not exist here, where there were 100 QTL on each chromosome. With a pedigree-based inbreeding constraint and genome-based estimated breeding values, genomic rates of inbreeding substantially exceeded the value of its constraint. In contrast, with a genome-based inbreeding constraint and genome-based estimated breeding values, marker frequencies changed, but this change was limited by the inbreeding constraint at the marker position.

**Conclusions:**

To control inbreeding, it is necessary to account for it on the same basis as what is used to estimate breeding values, i.e. pedigree-based inbreeding control with traditional pedigree-based BLUP estimated breeding values and genome-based inbreeding control with genome-based estimated breeding values.

## Background

Traditional pedigree-based BLUP (Best Linear Unbiased Prediction) estimated breeding values (EBV) [1] are based on pedigree information and recordings of selection candidates and relatives, e.g. sibs of candidates, as in aquaculture breeding schemes, where many traits (e.g. disease resistance and fillet quality) cannot be measured on the candidates. For genomic breeding values, the effects of dense genetic markers are first estimated in a test population and later used to predict breeding values of selection candidates [2]. Genome-based EBV, i.e. EBV based on high-density marker data across the genome, generally have higher accuracy than pedigree-based BLUP EBV, because genetic markers provide a more accurate relationship matrix than pedigree [3], which is based on expected genetic relationships. For example, the expected relationship between two full-sibs is 0.5 but markers show that the true relationship deviates from 0.5 [4] and varies among pairs of sibs, depending on the segregation of the parental chromosomes. However, the increased accuracy of genome-based EBV can differ between methods used to estimate them and, e.g., the number of genes affecting the trait. The genomic BLUP methodology has shown highest accuracy for traits without large quantitative trait loci (QTL) but the BayesB method has shown highest accuracy for traits with known large QTL, because it puts higher weight on genetic markers with large effects [5,6].

Optimum contribution selection [7,8] is a selection method that maximises genetic gain while restricting the rates of inbreeding in the progeny by restricting relationships between selected parents. Until now, pedigree-based relationship matrices have been used to control inbreeding rates, which constrain inbreeding rates at a neutral locus that is not linked to any QTL. It may be questioned whether such a locus exists, especially since genomic selection and other studies suggest that most traits are affected by a large number of QTL across the genome [9-12]. Thus, using genomic relationships may help to better control genome-based inbreeding, and may provide a tool for breeders to manage footprints of selection [13-16].

The aim of this study was to understand the consequences for genetic variability across the genome when genomic information is used to estimate EBV and in managing the inbreeding generated in the course of selection on genome-enhanced EBV. The consequences are measured by genetic gain and the pedigree- and genome-based rates of inbreeding and local inbreeding across the genome. Breeding schemes are compared by simulating truncation selection or optimum contribution selection with a restriction on pedigree- or genome-based inbreeding, and with selection on genome- or pedigree-based BLUP EBV. The trait under selection is a trait for which information on selection candidates comes from full-sibs, which provides a challenging test for developing theory, because the use of genome-based EBV is most beneficial in this situation [3]. In addition, these so-called sib-tests are commonly applied in practical breeding schemes.

## Methods

### Simulation of populations

A base population with an effective size of 1000 was simulated for 4000 generations. Details are described in [17]. One hundred sires and 100 dams from generation 4000 were randomly selected to create generation *G0*, consisting of 3000 selection candidates (*Ncand*) and 3000 or 6000 test sibs (*Ntest*). In later generations (*G1-G10*), selection was done by truncation or optimum contribution selection. Inbreeding coefficients based on pedigree (*F*_*ped*_) and rates of inbreeding based on pedigree (*ΔF*_*ped*_) assumed that the *G0* individuals were unrelated base individuals.

Simulation of the genomes has been described elsewhere [17]. The genome consisted of 10 pairs of chromosomes (1Morgan each). All polymorphisms were generated during the 4000 generations of the Fisher-Wright population model [18,19]. The infinite sites mutation model [20] was used to create new bi-allelic single nucleotide polymorphisms (SNP), using a mutation rate of 10^-8^ per nucleotide and assuming 10^6^ nucleotides per cM. Inheritance of the SNP followed Mendel’s law and the Haldane mapping function [21] was used to simulate recombination. One hundred SNP per chromosome were sampled randomly without replacement from SNP with a minor allele frequency (MAF) > 0.05 and used as QTL, i.e. the total number of QTL was 1000. From the remaining SNP, 1000 SNP with the highest MAF over all chromosomes were chosen as genetic markers. In addition, 100 artificial identical-by-descent (IBD) markers were positioned at equal distances on each chromosome. These IBD markers were not involved in selection, but were assigned unique founder alleles in generation *G0*, in order to monitor the increase of the local genomic IBD at these positions.

Additive effects of the QTL alleles were sampled from a gamma distribution with a shape parameter of 0.4 and a scale parameter of 1.66 [9]. The QTL effects were assumed to be either positive or negative with a probability of 0.5 because the gamma distribution only gives positive values. After sampling, these QTL effects were standardized so that the total genetic variance was 1.

### Calculation of phenotypic values and true and estimated breeding values

The true breeding value (*TBV*) of an individual was calculated as:

$$TBV_{i}=\sum_{j=1}^{1000}(x_{ij1}g_{j1}{+}_{\mathrm{xij}}g_{j2})$$

where *x*_*ijk*_ is the number of copies of the *k*^th^ allele that individual *i* has at the *j*^th^ QTL position and *g*_*jk*_ is the effect of the *k*^th^ allele at the *j*^th^ position. The phenotypic values, *y*_*i*_, of individuals in the sib-test were simulated by:

$$y_{i}=TBV_{i}+{ɛ}_{i}$$

where *ɛ*_*i*_ is an error term for animal *i*, which was normally distributed with mean zero and variance *σ*^*2*^_*e*_, which was adjusted so the heritability was 0.4.

Marker effects, *â*_*j*_, were predicted using the genome-based BLUP method described in [2], named GBLUP hereafter. The statistical model used was:

$$y_{i}=\mu +\sum_{j}^{n}X_{\mathrm{ij}}a_{j}+e_{i}$$

where *y*_*i*_ is the record of test sib *i*, *μ* is the overall mean, *n* is the total number of markers, *X*_*ij*_ denotes the standardised marker genotype, *a*_*j*_ is the random effect of the *j*^th^ marker and Var(*a*_*j*_) is assumed 1/*n* since the total genetic variance was standardised to 1, *e*_*i*_ is a random residual. *X*_*ij*_ was standardised to a mean of 0 and a variance of 1:

$$X_{\text{ij}}=-2p_{j}\text{/√}H_{j}$$

denotes that the individual is homozygous for the first allele; (1-2*p*_*j*_)/√*H*_*j*_ denotes that it is heterozygous; and (2-2*p*_*j*_)/√*H*_*j*_ denotes that it is homozygous for the second allele, where *H*_*j*_ is the marker heterozygosity and *p*_*j*_ is the frequency of the second allele. Division by √*H*_*j*_ results in every SNP explaining an equal amount of variance a priori (independent of the frequency of the SNP). The genetic variance explained by a SNP is *R*^2^*V_*QTL*_, where *R*^*2*^ is the linkage disequilibrium between the SNP and the nearest QTL, and *V*_*QTL*_ is the variance due to this QTL. Division by √*H*_*j*_, avoids making the assumption that SNP with high *H*_*j*_ have proportionally higher *R*^*2*^, which would especially be questionable in a situation where most QTL have a low frequency, as is the case when the QTL alleles are in mutation-drift equilibrium and even more so if the QTL are under selection. However, QTL with low allele frequencies are expected to have lower *V*_*QTL*_. Here, none of the markers used had low *H*_*j*_, thus correction of *X*_*ij*_ by √*H*_*j*_ is expected to affect the results only marginally.

Genome-based BLUP EBV (*GEBV*) were estimated by summing across the estimated effects of the markers:

$$GEBV_{j}=\sum_{j}^{n}X_{\mathrm{ij}}{\hat{a}}_{j}$$

In addition, *GEBV* were calculated with method BayesB, as described in [2]. BayesB uses the same statistical model as GBLUP but attempts to reduce the weight of SNP that are estimated to have no association with QTL. It also assumes a priori that many SNP have no effect and few SNP (1000 here) have t-distributed effects.

Traditional pedigree-based BLUP EBV (*TEBV*) were estimated with the method described in [1], named TBLUP hereafter, in which genetic relationships are based on pedigree information. Pedigree recording started in generation *G0*.

### Optimum contribution selection and mating

The optimum contribution selection algorithm of [7] was used, i.e. the genetic level of the next-generation animals, *g*_*t+1*_ = **c**_**t**_**’EBV**_**t**_ (**EBV**_**t**_ contains either *GEBV* or *TEBV*), was maximised, where **c**_**t**_ is a vector of genetic contributions of the selection candidates to generation *t* + 1. Rates of inbreeding were restricted by constraining the average relationship of the selection candidates to C¯t+1=ct'Atct/2, where **A**_**t**_ was a relationship matrix among the selection candidates, ${\bar{C}}_{t+1}=1-{\left(1-\Delta F_{d}\right)}^{t}$, and *ΔF*_*d*_ was the desired rate of inbreeding [8], i.e. 0.005 or 0.010 per generation. The relationship matrix was either based on pedigree or genomic data. For the latter, it equalled **G**_**t**_ = **X**_**t**_**X**_**t**_**’**/*n*[3].

Having calculated the optimum contribution vector **c**_**t**_, the next generation of offspring were produced by sampling a male and a female parent with replacement, according to the probabilities given by 2**c**_**t**_, which resulted in random mating. One hundred full-sib families were created each generation, from *G1* to *G10*. Each family was split into 30 selection candidates and 30 or 60 test sibs. The test sibs were recorded for the trait.

### Recording

For the schemes using *TEBV*, test sibs were only phenotyped, while for those using *GEBV*, test sibs were phenotyped and genotyped to estimate the SNP effects and selection candidates were genotyped. This sib-test provided a challenging test for the management of genetic variation but it is also very relevant as it in such circumstances that the use of genomic data in breeding value estimation is of greatest value.

### Truncation selection and mating

For a simple comparison of TBLUP and GBLUP, truncation selection was used instead of optimum contribution selection. Each generation, 100 sires and 100 dams were selected from 3000 selection candidates on their breeding values estimated either from TBLUP or GBLUP. These sires and dams were pair-wise mated to produce 100 full-sib families for the next generation, using sampling without replacement.

### Calculation of genomic identity-by-descent

Genomic IBD was obtained by calculating the allele frequencies of the founder alleles at the IBD markers, i.e. *f*_*ij*_ for founder allele *j* at IBD marker *i*. Their homozygosity, i.e. probability of IBD was then calculated for IBD marker *i* as Σ_j_*f*_*ij*_^*2*^. To calculate *ΔF*_*IBD*_, this IBD probability was averaged over all IBD markers to evaluate the overall IBD over the genome in generations*G0* to *G10*. IBD profiles differed between replicates because the position and size of the QTL differed.

### Statistics

TBLUP and GBLUP schemes were compared using either truncation selection or optimum contribution selection. In the latter case, the constraint was based on relationships derived either from pedigree (*ΔF*_***A***_) or from markers (*ΔF*_***G***_). The schemes were run for ten generations (*G1*-*G10*) and summary statistics for each of the schemes were based on 100 replicated simulations. The breeding schemes were compared for rates of inbreeding per generation (*ΔF*) and genetic gain (*ΔG*), expressed in genetic standard deviation units of generation *G0* (σ_*a*_) in generation *G10*. The rates of inbreeding per generation were calculated in two ways, either from the pedigree *ΔF*_*ped*_ or using the IBD loci *ΔF*_*IBD*_*.* Here, the symbol *ΔF*_*A*_ denotes a constraint on pedigree-based relationships and thus on inbreeding, and *ΔF*_*ped*_ denotes the rate of inbreeding as calculated from the pedigree that results from the breeding scheme. Inbreeding coefficients at each IBD locus were also stored to analyse inbreeding rates as a function of locus and generation.

## Results

### Truncation selection with TBLUP and GBLUP breeding values

**Figure 1:**
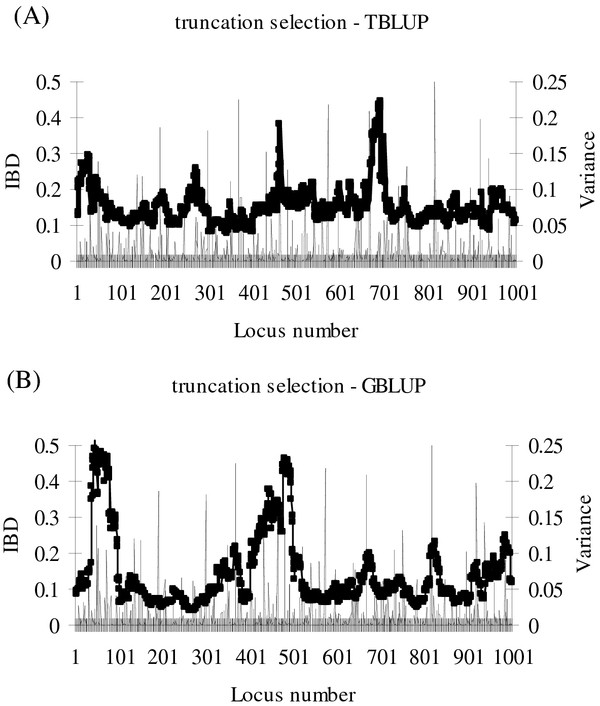
**Identity-by-Descent for one replicate of truncation selection on breeding values estimated by TBLUP or GBLUP.** Variance (σ_*g*_^*2*^-unit) explained by QTL in generation *G0* () and Identity-by-Descent (IBD;) in generation *G10* with truncation selection and (**A**) TBLUP (BLUP based on pedigree relationships) or (**B**) GBLUP (BLUP based on genomic relationships); results are from one replicate with 3000 selection candidates and 3000 test sibs.

The truncation selection schemes evaluated constitute classical selection with comparisons made at constant selection intensity and scheme size. For these schemes, genetic gain was 11% higher for GBLUP than for TBLUP and, although *ΔF*_*ped*_ was much lower for GBLUP than TBLUP, *ΔF*_*IBD*_ was only slightly lower for GBLUP than TBLUP (Table 1). Rate of inbreeding measured by *ΔF*_*IBD*_ was 51% greater than inbreeding measured by *ΔF*_*ped*_ for TBLUP schemes but 292% greater for GBLUP schemes (Table 1). Thus, with GBLUP schemes the increase in genomic inbreeding was well above the increase in pedigree inbreeding. A higher genome-wide IBD profile was obtained with TBLUP than with GBLUP (Figure 1). These results show the importance of considering the basis for constraining rates of inbreeding.

**Table 1:** Truncation selection on breeding values estimated using TBLUP or GBLUP

**Breeding value estimation**	**ΔG (se)**	**ΔF**_**ped**_**(se)**	**ΔF**_**IBD**_**(se)**
TBLUP	2.49 (0.035)	0.0156 (0.0001)	0.0235 (0.0009)
GBLUP	2.77 (0.026)	0.0053 (0.0002)	0.0209 (0.0005)

Genetic gain (*Δ*G), rate of inbreeding based on pedigree (*Δ*F_ped_) or on genomic IBD (*Δ*F_IBD_) relationship matrices at generation *G10* with truncation selection and either TBLUP or GBLUP breeding value estimates^a^.

^a^Number of test sibs = 3000; number of selection candidates = 3000.

### Optimum contribution selection with TBLUP and GBLUPEBV

Table 2 shows that, with the current practice of explicitly constraining *ΔF*_***A***_*,* the desired rate of inbreeding, *ΔF*_*d*_, was observed in *ΔF*_*ped*_ but not when the constraint was implemented based on *ΔF*_***G***_. Table 2 also shows that when selection was on TBLUP, the observed *ΔF*_*IBD*_ substantially exceeded *ΔF*_*d*_.

**Table 2:** Optimum contribution selection on breeding values estimated using TBLUP or GBLUP

**Ntest**	**ΔF**_**d**_	**ΔG (se)**	**ΔF**_**ped**_**(se)**	**ΔF**_**IBD**_**(se)**
		**ΔF**_**A**_**constraint – GBLUP**		
3000	0.005	3.08 (0.035)	0.0050 (0.0001)	0.0211 (0.0004)
6000	0.005	3.10 (0.035)	0.0048 (0.0001)	0.0226 (0.0004)
6000	0.010	3.31 (0.037)	0.0098 (0.0003)	0.0422 (0.0008)
		**ΔF**_**G**_**constraint – GBLUP**		
3000	0.005	1.91 (0.026)	0.0041 (0.0001)	0.0051 (0.0001)
6000	0.005	1.95 (0.024)	0.0039 (0.0001)	0.0053 (0.0001)
6000	0.010	2.41 (0.028)	0.0071 (0.0002)	0.0102 (0.0002)
		**ΔF**_**A**_**constraint – TBLUP**		
3000	0.005	2.26 (0.003)	0.0050 (0.0001)	0.0068 (0.0001)
6000	0.005	2.50 (0.003)	0.0049 (0.0001)	0.0074 (0.0001)
6000	0.010	2.63 (0.003)	0.0102 (0.0002)	0.0151 (0.0003)
		**ΔF**_**G**_**constraint – TBLUP**		
3000	0.005	1.41 (0.041)	0.0193 (0.0004)	0.0121 (0.0002)
6000	0.005	1.44 (0.039)	0.0185 (0.0004)	0.0122 (0.0002)
6000	0.010	1.48 (0.046)	0.0300 (0.0008)	0.0183 (0.0003)

Genetic gain (*ΔG*), rate of inbreeding based on pedigree (*ΔF*_*ped*_) and on genomic IBD (*ΔF*_*IBD*_*)* relationship matrices at generation *G10* when the constraint on relationship was either pedigree-based (*ΔF*_*A*_) or marker-based (*ΔF*_*G*_) with TBLUP or GBLUP breeding value estimates^a^.

^a^*Ntest* = number of test sibs; ΔF_d_ = desired rates of inbreeding; number of selection candidates = 3000.

When *ΔF*_*A*_ was constrained, *ΔG* was substantially greater with GBLUP than with TBLUP, by ~ 35% when *Ntest* = 3000 and by ~25% when *Ntest* = 6000. Due to the inadequacy of constraining *ΔF*_***A***_, *ΔF*_*IBD*_ increased above *ΔF*_*d*_ even more strongly with GBLUP than with TBLUP (by ~320-360% with GBLUP and ~35-50% with TBLUP). The greatest increase in *ΔFIBD* was observed when *Ntest* = 6000.

When *ΔF*_***G***_ was constrained, *ΔG* was again greater with GBLUP than with TBLUP, however in this case, the magnitudes of the increase depended on *ΔF*_*d*_, i.e. by ~35% when *ΔF*_*d*_ was set at 0.005 and *Ntest* = 3000 and by ~65% when *ΔF*_*d*_ was set at 0.010. When applying the constraint using *ΔF*_***G***_ with TBLUP, *ΔF*_*IBD*_ substantially exceeded the desired *ΔF*_*d*_. The observed *ΔF*_*ped*_ was even more extreme, and was ~50% greater than *ΔF*_*IBD*_*.* However, when applying the constraint using *ΔF*_***G***_ with GBLUP, *ΔF*_*ped*_ was ~70-80% of the desired *ΔF*_*d*_*.*

**Figure 2:**
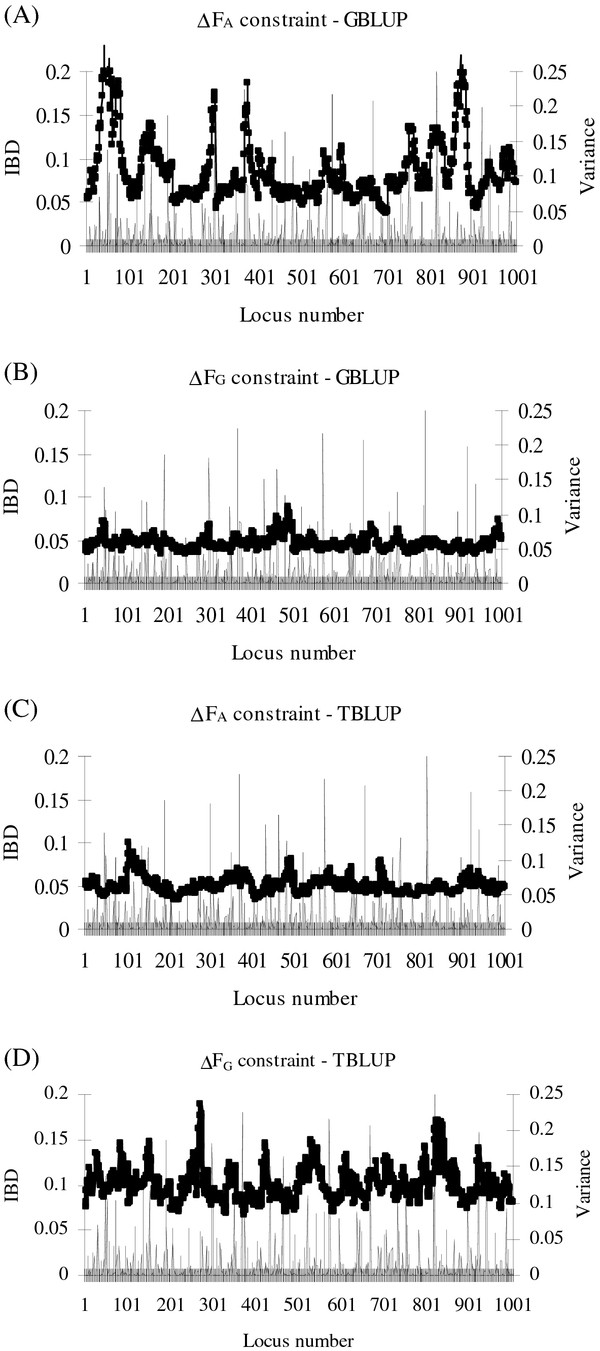
**Identity-by-Descent for one replicate of optimum contribution selection on breeding values estimated by TBLUP or GBLUP.** Variance (σ_*g*_^*2*^-unit) explained by QTL in generation *G0* () and Identical-by-descent (IBD;  ) in generation *G10* with Δ*F*_*A*_ constraint based on pedigree relationships and GBLUP (BLUP based on genomic relationships) (**A**), Δ*F*_*G*_ constraint and GBLUP (**B**), Δ*F*_*A*_ constraint and TBLUP (BLUP based on pedigree relationships) (**C**) or Δ*F*_*G*_ constraint based on marker relationships and TBLUP (**D**); results are from one replicate with 3000 selection candidates and 3000 test sibs and a desired rate of inbreeding of 0.005.

With GBLUP, schemes that constrained *ΔF*_***A***_ showed a substantially more variable IBD profile across the genome (Figure 2A), than those that constrained *ΔF*_***G***_ (Figure 2B). In contrast, with TBLUP, schemes that constrained *ΔF*_***A***_ showed little variation in the genome-wide IBD profile (Figure 2C), while those that constrained *ΔF*_***G***_ showed a very erratic profile (Figure 2D).

**Figure 3:**
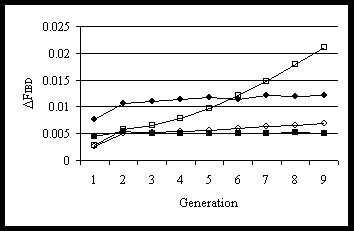
**Rates of genomic inbreeding over generations.** Rates of inbreeding based on genomic IBD (Δ*F*_*IBD*_) over generations for schemes with Δ*F*_*A*_ constraint based on pedigree relationships and GBLUP (BLUP based on genomic relationships)(□), Δ*F*_***G***_ constraint based on genomic relationships and GBLUP (▪), Δ*F*_***A***_ constraint and TBLUP (BLUP based on pedigree relationships) (_°_) or Δ*F*_***G***_ constraint and TBLUP (·); results are from the scheme with 3000 selection candidates and 3000 test sibs and a desired rate of inbreeding of 0.005.

Schemes that constrained *ΔF*_***G***_ showed a constant *ΔF*_*IBD*_ over generations but at a higher level than the constraint when selection was on TBLUP (Figure 3). Schemes that constrained *ΔF*_***A***_ showed an increase in *ΔF*_*IBD*_ over generations, in particular when selection was on GBLUP. This increase in *ΔF*_*IBD*_ over generations is probably due to fixation of favourable alleles, which occurs faster with GBLUP. This increased *ΔF*_*IBD*_ over time also suggests that the constraint on *ΔF*_***A***_ becomes less restrictive over time when selection uses *GEBV*.

**Figure 4:**
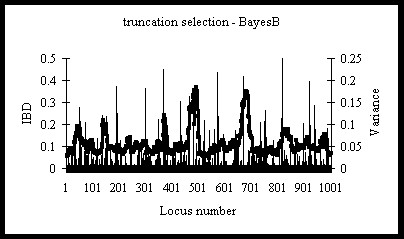
**Identity-by-Descent for one replicate of truncation selection on breeding values estimated by BayesB.** Variance (σ_g_-unit^2^) explained by QTL in generation *G0* () and IBD () in generation *G10* with truncation selection and BayesB estimated breeding values; results are from one replicate with 3000 selection candidates and 3000 test sibs.

Use of *GEBV* derived using BayesB showed very similar results as using *GEBV* from GBLUP in terms of *ΔG* and accuracy of selection (Table 3). BayesB had significantly higher *ΔF*_*IBD*_ (0.0235 compared to 0.0209), which can be explained by a larger focus on some SNP, which increased local IBD values but also the overall *ΔF*_*IBD*_. These results are in concordance with [5], who found that BayesB is advantageous for traits with a few large QTL and many smaller QTL. Here, 1000 QTL were simulated, which disadvantaged BayesB compared to GBLUP. With BayesB, the IBD profile had a few IBD peaks but was generally quite uniform (Figure 4).

**Table 3:** Truncation selection on breeding values estimated using GBLUP or BayesB

**Breeding value estimation**	**ΔG (se)**	**ΔF**_**ped**_**(se)**	**ΔF**_**IBD**_**(se)**
GBLUP	2.77 (0.026)	0.0053 (0.0002)	0.0209 (0.0005)
BayesB	2.73 (0.027)	0.0053 (0.0001)	0.0235 (0.0005)

Genetic gain (*Δ*G) and rate of inbreeding based on pedigree (*ΔF*_*A*_) or on genomic IBD (*ΔF*_*G*_) relationship matrices at generation *G10* with truncation selection and either GBLUP or BayesB breeding value estimates^a^.

^a^Number of test sibs = 3000; number of selection candidates = 3000.

## Discussion

Several methods for the management of *ΔF*_*A*_ have been suggested in the literature [7,8,13,14,16]. With the development of genomic selection, genomic relationships have become available in addition to the traditional pedigree-based relationships. An immediate question then is whether the constraint should be based on pedigree (*ΔF*_***A***_) or genomic (*ΔF*_***G***_) relationships, when combined with breeding value estimation based on pedigree (TBLUP) or genomic (GBLUP) information. Our results address this question by showing that if the information sources used to estimate breeding values and to constrain *ΔF* are identical, i.e. either both pedigree-based or both genomic-based, then the resulting rates of genomic inbreeding correspond to the desired values and the rates are reasonably uniform across the genome. However, if different information is used to calculate breeding values and to constrain *ΔF*, e.g., the EBV are based on genomic information and the *ΔF* constraint is on pedigree information, the resulting rate of inbreeding, based on *ΔF*_*IBD*_, is much higher than expected.

It is important to recognise the distinction between the three relationships measures between two individuals *u* and *v* that are considered in this study: *a*_*uv*_, the numerator relationship derived from pedigree; *g*_*uv*_, the identity by state relationship obtained from the markers accumulated over time and used to estimate *GEBV*; and *t*_*uv*_, the relationship between the identity-by-descent markers defined in the base population used for reference. EBV were estimated either using *a*_*uv*_ (TBLUP) or *g*_*uv*_ (GBLUP) and rates of inbreeding were controlled using either *a*_*uv*_ or *g*_*uv*_*,* since these values are accessible and known. However, the examination of the development of relationships over time is based on *a*_*uv*_ or *t*_*uv*_ since these measure IBD with respect to the base generation *G0*, with the objective of controlling the drift in an unknown locus from the start of selection. Using *a*_*uv*_ for this control will be predictive and unbiased for a neutral locus that is unlinked to variants with an effect, whereas *t*_*uv*_ is an empirical value that is not restricted by neutrality or position of the locus. Using *g*_*uv*_ to control inbreeding has the advantage of acting upon relationships that already exist in generation *G0*, whereas both *a*_*uv*_ and *t*_*uv*_ assume that *G0* animals are unrelated.

Interpretation of the consequences of these differences in breeding value estimation, control of inbreeding and assessment of IBD may be helped by considering the impacts when *u* and *v* are full-sib candidates. For the simulated sib-tested trait, the distinctions between EBV estimated using TBLUP versus GBLUP are clear, since *u* and *v* will have the same EBV with TBLUP but information on Mendelian sampling terms to differentiate the sibs is available with GBLUP. For the same reason, full-sibs *u*, *v* and *w* will be treated as having equal pair-wise relationships when measured using *a*_*uv*_ but different pair-wise relationships when measured using *g*_*uv*_.

The incentive to include genomic data into breeding schemes comes from its use in breeding value estimations. With GBLUP, the results of Table 2 are interpretable from the framework outlined in the previous paragraph. When *ΔF*_*A*_ is constrained, the relationship of two selected individuals *u* and *v* will be greater than their pedigree relationship since their high merit implies that on average they are more likely to share QTL, as well as flanking segments that, over time, will tend to become more homozygous along with the QTL. Thus the genome will contain segments where segregation is not free from the influence of selection and where there is more homozygosity than predicted by *a*_*uv*_ and so E[*t*_*uv*_|*a*_*uv*_] > *a*_*uv*_, i.e. when we condition on or constrain *a*_*uv*_, we expect *t*_*uv*_ to exceed *a*_*uv*_. Hence, constraining *ΔF*_***A***_ will result in underestimating *ΔF*_IBD_. The extent of this underestimation will depend on the density of the QTL across the genome and the linkage disequilibrium between the QTL. In a similar way, when *ΔF*_***G***_ is constrained, E[*a*_*uv*_|*g*_*uv*_] < *g*_*uv*_, and so *ΔF*_ped_ will be less than the target value, whereas E[*t*_*uv*_|*g*_*uv*_] ~ *g*_*uv*_ in this context.

If breeding value estimation is based on TBLUP then truncation selection will select whole full-sib families but not with selection with optimized contributions. When inbreeding management is based on *ΔF*_*A*_, then E[*t*_*uv*_|*a*_*uv*_] > *a*_*uv*_ after selection, for the same reasons as before, i.e. large segments are not free of the influence of selection, and *ΔF*_*IBD*_ is greater than the desired rate of inbreeding. With TBLUP, the impact on *ΔF*_*IBD*_ is not as large as when using GBLUP since the QTL are not identified as accurately and response to selection is less. The most challenging outcomes occur when *ΔF*_***G***_ is constrained. In this case, two full-sibs might be selected if they appear less related based on markers than expected based on pedigree. On the one hand, E[*t*_*uv*_|*g*_*uv*_] is greater than *g*_*uv*_ since the prediction errors of *t*_*uv*_ from *g*_*uv*_ will be positive because variation not explained by *g*_*uv*_ is more likely to reflect the full-sib pedigree relationship. On the other hand, E[*a*_*uv*_|*g*_*uv*_] will be much greater than *g*_*uv*_ since the genomic relationships of those sibs that are selected will be below average and thus lower than *a*_*uv*_. Hence, *ΔF*_*ped*_ will be greater than *ΔF*_*IBD*_*.* It is notable that the combination of TBLUP with *ΔF*_***G***_ delivers the least gain for close to the highest rates of inbreeding, by either measure (see Table 2). Although this combination may not be practical, it is instructive.

The dynamics of *ΔF*_*IBD*_ over time can also be explained within this framework. The only combination without a stable trend in inbreeding was when *ΔF*_***A***_ was used as the constraint with GBLUP; all other combinations showed a stable *ΔF*_*IBD*_ (Figure 3). With GBLUP and *ΔF*_*A*_, *ΔF*_*IBD*_ continuously increased during generations *G1* to *G10* (i.e. the rate of inbreeding increased), probably because the estimates of marker effects are more persistent over generations than the pedigree relationships, implying that changes across many generations of the frequencies of (selected) chromosome segments are not picked up by pedigree relationships. As a result, GEBV favour specific chromosome segments generation after generation, thereby increasing *ΔF*_*IBD*_ but not *ΔF*_*ped*_. Thus, especially in the longer term, discrepancies in the information used to estimate the breeding values and to control inbreeding will hamper the control of inbreeding.

Some of the outcomes observed here depend on the number and distribution of effects of the QTL: we simulated 1000 QTL on a genome of 10 M. With a much smaller number of QTL, i.e. when the genome is predominantly composed of neutral loci that are weakly linked to QTL, *ΔF*_*IBD*_ is expected to be closer to *ΔF*_*ped*._ However, increasing evidence from well-studied traits such as human height indicates that many traits are composed of many QTL each explaining a small part of the variance [12]. Trait heritability was rather high here (0.4). With a lower trait heritability, the number of sib-tested animals needs to be increased in order to obtain similar accuracies as found in the present study [22].

Inbreeding at QTL positions is desirable in breeding schemes because this increases the frequency of the positive alleles towards homozygosity. However, narrowing the genomic IBD peak at the QTL positions is also desired, so that the remaining genome is as little as possible affected by selection at individual QTL, i.e. the footprint of selection should be as small as possible. Based on the hitchhiking effect [23], a broad genomic IBD peak around the largest QTL would have been expected. However, when GBLUP was used and *ΔF*_*G*_ was constrained, the genome-wide IBD profile was rather flat (Figure 1B), which suggests that this selection method spreads the selection pressure quite evenly over many loci in order to control *ΔF*_*IBD*_. This implies that, in practical breeding schemes, it is not necessary to implement additional constraints on the genomic inbreeding at positions surrounding large QTL in order to avoid excessive rates of genomic inbreeding in QTL regions.

A reduction of the footprint of selection may also be achieved by increasing the frequencies of a broad spectrum of QTL alleles slowly, instead of heavily selecting on the biggest QTL with the danger of a large selection footprint. The latter seems to have occurred with genomic optimum contribution selection with estimation based on GBLUP, since the increase of the IBD was rather flat across the genome for that scenario (Figure 1B). The GBLUP method assumes that all SNP explain the same genetic variance (1/*n*, i.e. 5000 here). Use of BayesB resulted in an IBD profile with more peaks (Figure 4) but not to the extent that local restrictions of inbreeding would be required. Further research is needed to investigate whether in situations with a few large QTL and using BayesB, directed measures would be required to reduce the footprint of selection in the regions of large QTL, and how such measures should be implemented. In case the SNP effects are estimated accurately and do not change over time, a model that maximises the genetic gain over a specified time horizon can also be used [3], since this will spread the selection intensity optimally across the genomic regions in order to maximise long-term genetic gain.

In our study, allelic effects were assumed purely additive, whereas dominance and higher order interactions may occur. Dominance interactions can be partly accommodated by including a regression on (genomic) inbreeding in the model used to estimate breeding values. This will correct the (G)EBV so that they are valid for matings that result in non-inbred offspring. In practice, animals are expected to be mated to related selection candidates and thus their (G)EBV should be corrected for the inbreeding depression times their expected future inbreeding, which is their average coancestry with the selection candidates [24].

The design of the breeding scheme studied here resembles that of aquaculture breeding schemes, which rely heavily on sib-testing. In this design, traditional selection relies only on family information and either selects entire full-sib families or rejects them. The use of genomic selection makes it possible to estimate within-family deviations, and thus to distinguish between family members. This sib-testing design may have exaggerated the differences between genomic and traditional selection, and their effects on genome structure, because in most practical breeding schemes traditional selection also yields an estimate of the within-family deviation. However, in such schemes, genomic selection will estimate within-family deviations more accurately and thus the general outcomes of our study will still hold. In addition, genomic selection will be applied mainly in situations in which traditional selection yields little or no information on the within-family genetic component.

This study shows that serious interactions can occur between the methods used to estimate breeding values and the types of relationships used to control inbreeding. Results showed that outcomes are most stable and predictable, i.e. the final outcome reflects the constraint, when the same information is used to estimate breeding values and to control inbreeding. Thus, genomic selection has to be combined with genomic control of inbreeding in order to effectively manage *ΔF*_*IBD*_.

## Conclusions

Desired control of inbreeding was only achieved when it was managed using the same information as is used to estimate breeding values, i.e. pedigree-based inbreeding control with pedigree-based estimation of breeding values and genome-based inbreeding control with genome-based estimation of breeding values. In addition, the genome-based estimation of breeding values allows management of changes in genomic inbreeding, and thus changes in pedigree-based inbreeding are probably no longer relevant.

## Competing interests

The authors declare that they have no competing interest.

## Authors’ contributions

AKS wrote the main computer program, ran computer programs and drafted the manuscript. JAW contributed in setting up the study, interpreting results and writing the manuscript. THEM wrote computer modules for genome-wide breeding value estimation and for Fisher-Wright populations. All authors have approved the final manuscript.
